# Optimal Intervention for Initial Treatment of Anastomotic Biliary Complications After Right Lobe Living Donor Liver Transplantation

**DOI:** 10.3389/ti.2022.10044

**Published:** 2022-04-22

**Authors:** Min Seob Kim, Suk Kyun Hong, Hye Young Woo, Jae-Hyung Cho, Jeong-Moo Lee, Kyung Chul Yoon, YoungRok Choi, Nam-Joon Yi, Kwang-Woong Lee, Kyung-Suk Suh

**Affiliations:** Department of Surgery, Seoul National University College of Medicine, Seoul, South Korea

**Keywords:** living donor liver transplantation, anastomotic biliary complications, endoscopic retrograde cholangiography, percutaneous transhepatic biliary drainage, right anterior hepatic duct, right posterior hepatic duct

## Abstract

**Background:** This study evaluated endoscopic retrograde cholangiopancreatography (ERCP) and percutaneous transhepatic biliary drainage (PTBD) as interventions for patients with anastomotic biliary complications (ABC) after living donor liver transplantation (LDLT).

**Methods:** Prospectively collected data of patients who were diagnosed with ABC after LDLT between January 2013 and June 2017 were retrospectively reviewed.

**Results:** There were 57 patients who underwent LDLT with a right liver graft using duct-to-duct biliary reconstruction and experienced ABC. Among the patients with RAD involvement, there were no significant differences in the intervention success (*p* = 0.271) and patency rates (*p* = 0.267) between ERCP and PTBD. Similarly, among the patients with RPD involvement, there were no significant differences in the intervention success (*p* = 0.148) and patency rates (*p* = 0.754) between the two procedures. Graft bile duct variation (*p* = 0.013) and a large angle between the recipient and graft bile duct (R-G angle) (*p* = 0.012) significantly increased the likelihood of failure of ERCP in the RAD. When the R-G angle was greater than 47.5°, the likelihood of ERCP failure increased.

**Conclusion:** We recommend PTBD when graft bile duct variation is presented in patients with RAD involvement and/or when the R-G angle is greater than 47.5°.

## Graphical Abstract

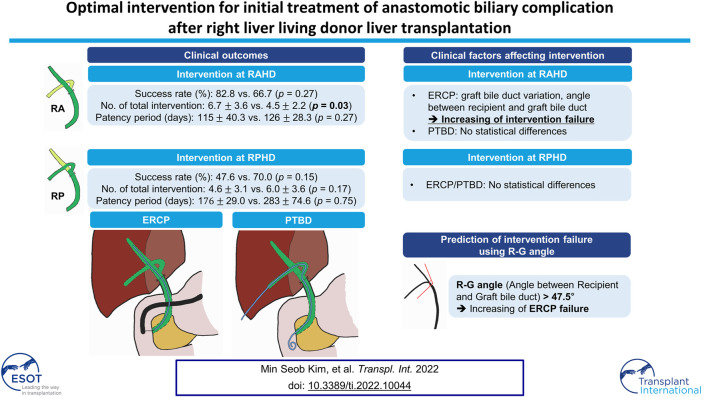

## Introduction

Liver transplantation (LT) with duct-to-duct (DD) biliary reconstruction has several physiologic advances and is a lifesaving treatment for patients with end-stage liver disease and hepatocellular carcinoma (1). In Asia, living donor liver transplantation (LDLT) is performed more often than deceased liver transplantation due to a shortage of cadaveric organ donors (2–4). Biliary anastomotic strictures or leakage are the most common complications following LT (5). LDLT is more susceptible to anastomotic biliary complications (ABC) compared to deceased liver transplantation (4, 6), because the right hemi-liver (RL) graft bile duct is short, arises at an acute angle, and has multiple openings that are prone to peribiliary vascular plexus damage. Interventional treatment is recommended for patients with ABC following LDLT with DD biliary reconstruction, because it is effective, non-invasive, and more convenient than surgery (7, 8). Endoscopic retrograde cholangiopancreatography (ERCP) is the first-line treatment, and percutaneous transhepatic biliary drainage (PTBD) may be performed as a rescue treatment if endoscopic treatment is unsuccessful (1, 3, 8, 9).

Anatomical variations of the RL graft bile duct influence the outcomes of DD biliary reconstruction (1, 4–6). The RL bile duct may have one or two duct openings, and a recent study by You *et al.* (4) recommended bilateral drainage for each of the right anterior and posterior hepatic ducts (RAD and RPD, respectively) of patients with ABC after LDLT with a RL graft, to improve final outcomes. There are limited studies examining which intervention (ERCP or PTBD) is more superior for each duct (RAD or RPD), with several factors affecting the success of ERCP or PTBD in either duct. Selection of the first-line treatment is particularly important because these patients have to undergo multiple consecutive procedures; therefore, the first-line treatment must be safe and convenient.

This study compared the efficacy of ERCP and PTBD in patients with ABC in the RAD or RPD after LDLT with a RL graft. We examined the factors that should be considered when selecting an intervention as a first-line treatment.

## Materials and Methods

### Study Design and Population

**FIGURE 1:**
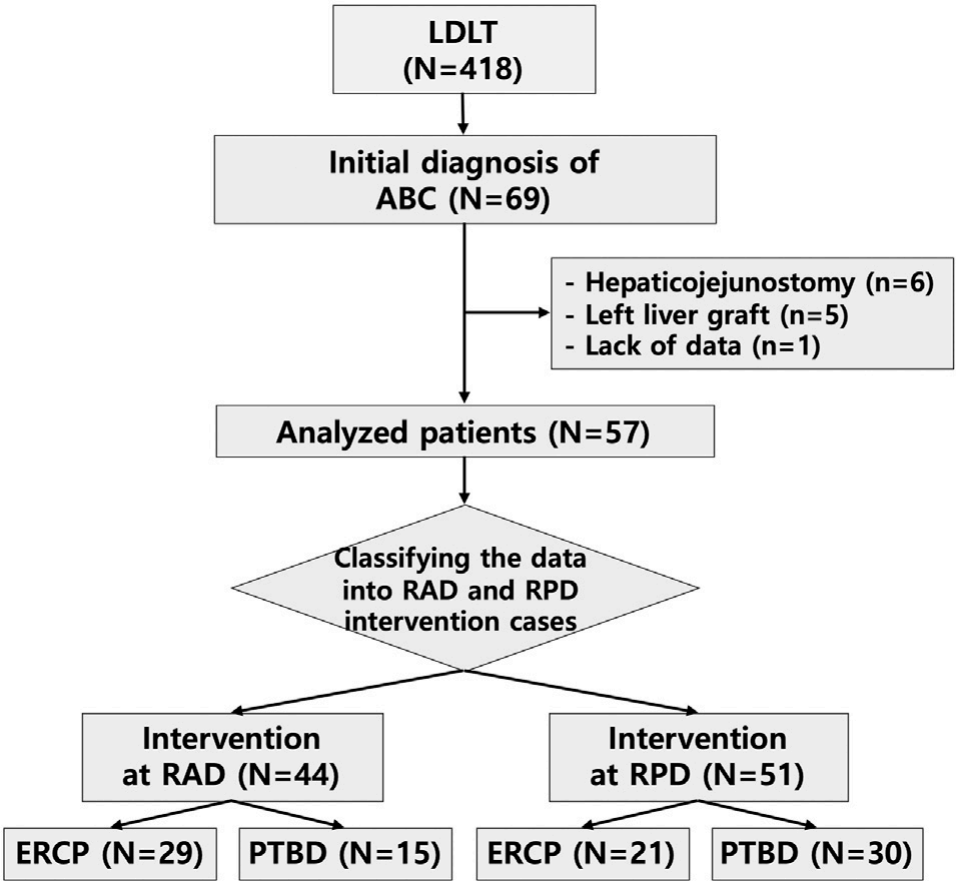
The employed process for patient exclusion and data classification. LDLT, living donor liver transplantation; ABC, anastomotic biliary complications; RAD, right anterior hepatic duct; RPD, right posterior hepatic duct; ERCP, endoscopic retrograde cholangiopancreatography; PTBD, percutaneous transhepatic biliary drainage.

This study included 418 patients who underwent LDLT at Seoul National University Hospital (SNUH) between January 2013 and June 2017. Sixty-nine patients (69/418, 16.5%) were newly diagnosed with ABC, such as anastomotic biliary stricture, anastomotic leakage, and anastomotic stricture with leakage, after LDLT, and these patients underwent either the ERCP or PTBD intervention initially. Among the patients with ABC, 12 were excluded because of hepaticojejunostomy biliary reconstruction (*n* = 6), a left liver graft (*n* = 5), and lack of data (*n* = 1). The demographic and baseline characteristics of the remaining 57 patients were analyzed. Among the 57 patients who were diagnosed with biliary complications, six patients underwent intervention only for the RAD, 13 patients underwent intervention only for the RPD, and 28 patients underwent intervention for both the RAD and RPD. Overall, 44 RAD interventions and 51 RPD interventions were performed, including both ERCP and PTBD procedures (Figure 1).

This study was approved by the Institutional Review Board of SNUH (approval no. 2101-132-1190). The requirement for informed consent was waived because of the study’s retrospective design. Data were retrospectively collected from medical records and reviewed. No organs from executed prisoners were used.

### Biliary Anastomosis

When performing DD biliary anastomosis, anastomoses between the graft hepatic duct and recipient bile duct were performed in an end-to-end fashion. A mixed interrupted and continuous suturing technique was performed using 6–0 absorbable suture material. The tailored telescopic reconstruction method (TTR) (10) was selected and performed intraoperatively according to the operator. In the case of TTR, the graft hepatic duct was anastomosed to the inner layer of the recipient bile duct with good vascularity. The shape of the anastomosis was similar to that of a telescope. The posterior and anterior walls were sutured continuously with 6–0 non-absorbable suture material. During anastomosis, if the graft bile duct opening was in the form of binoculars or the distance across the bile duct opening was short, one biliary anastomosis was performed according to the operator.

### Diagnosis of Anastomotic Biliary Complications

All patients received routine postoperative care according to the SNUH protocol. Inpatients and outpatients were assessed periodically, and liver computed tomography (CT) or magnetic resonance imaging (MRI) was performed when clinical symptoms, such as jaundice, itching, and abdominal pain, or abnormal laboratory findings, such as liver enzyme elevation and hyperbilirubinemia, were elicited. ABC was diagnosed in the presence of upstream bile duct dilatation or bile leakage in the anastomosis site.

### Management of Anastomotic Biliary Complications

Patients with ABC after LDLT were initially managed with supportive medical care. The intervention was selected with a multidisciplinary approach based on the patient’s history and clinical and laboratory findings. For patients diagnosed with biliary complications after LDLT, a multidisciplinary team, including the transplant and radiology teams, discussed the treatment plan together. If CT or MRI was performed in cases where biliary complications were suspected, the more appropriate intervention was determined based on the imaging findings. The findings we considered included the size of peripheral bile duct dilatation, angulation of the anastomosis site, and the possibility of percutaneous access.

### Endoscopic Retrograde Cholangiopancreatography

ERCP was performed under sedation. The side-view endoscope was inserted into the duodenum to check the ampulla of Vater, then cannulation was performed. If cannulation failed several times, papillotomy was performed using a needle knife. After cannulation, a guidewire was inserted and passed through the ABC. If anastomotic stricture was found through fluoroscopy, 4–10 mm sized balloon dilatation was performed. Plastic stents were inserted, with sizes 7, 8.5, and 10 F, and lengths between 5 and 15 cm, along the guidewire that had been passed through the ABC. In some cases, either a straight or pig tail catheter was selected according to the interventionist.

If the stent insertion through ERCP was successful, recurrence of biliary complications and procedure-related complications were not expected; thus, outpatient follow-up was performed after three to 6 months. After follow-up, it was decided whether to perform planned internal stent removal or revision. Further stricture site dilatation was optionally performed when there was no improvement in the patient’s biliary complications after initial stent insertion.

### Percutaneous Transhepatic Biliary Drainage

PTBD was performed by radiologic interventionists under local anesthesia. When a biliary stricture site was confirmed through fluoroscopy, 4–6 mm balloon dilatation was carried out after passage of the guidewire. After that, an 8.5 F pigtail catheter was inserted initially. For planned gradual dilatation, the catheter was extended from size 10 up to 14 F every 2–3 days while maintaining external PTBD. When the catheter was expanded to its maximum size, external PTBD was maintained for approximately 1 month and follow-up was performed at the outpatient clinic. Finally, when the patient’s symptoms remained stable, replacement with an internal stent was performed. The size and diameter of the internal stent were similar to those of the ERCP plastic stent.

The rendezvous method was also considered when the angle of the anastomosis site was acute or twisted, making it difficult to insert an internal stent through PTBD. If replacement with an internal stent was not possible due to tight biliary stricture, ERCP was re-tried while maintaining external biliary drainage. In addition, if it was determined that the biliary stricture could not be replaced by internal drainage, hepaticojejunostomy was performed in consideration of the patient’s quality of life. Similarly, an 8.5 or 10 F PTBD catheter was inserted in a place with biliary leakage to cover the site. External drainage was continued until the patient’s symptoms and radiologic findings improved.

### Definition

ABC was classified as stricture only, leakage only, and both stricture and leakage. Procedural success in strictures was defined by the ability to pass a catheter or stent through the anastomotic stricture site, which resulted in the improvement of clinical symptoms and/or laboratory findings during the hospitalization period. Procedural success in leakages was defined by the ability to cover the anastomotic leakage site with a catheter or stent. The overall success rate was defined as the ratio between the number of successful interventions and total number of interventions.

**FIGURE 2:**
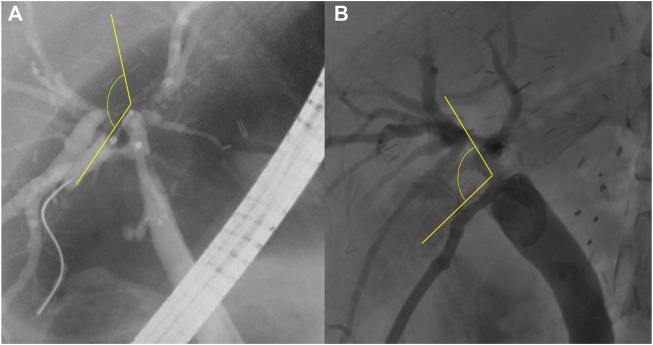
The angle between the recipient and graft bile ducts (R-G angle). The R-G angle is measured as the angle between the two straight yellow lines, shown on fluoroscopic imaging **(A)** during endoscopic retrograde cholangiopancreatography and **(B)** during percutaneous transhepatic biliary drainage.

Patency was defined as the period from the first intervention performed for initial biliary complications to the second intervention performed to treat recurred biliary complications. If the first intervention was performed over several days, the patency rate was defined as the period from the last day of the planned first intervention until the recurrence of complications. The angle between the recipient and graft bile ducts (R-G angle) was defined as the angle formed by the passage of the catheter or stent between the recipient and graft bile ducts on fluoroscopic imaging during ERCP or PTBD (Figure 2).

### Statistical Analysis

Categorical variables were presented as numbers and percentages, whereas continuous variables were presented as mean ± standard deviation. Categorical variables were compared using the χ^2^ test, Fisher’s exact test, and linear-by-linear association, and continuous variables were compared using the Student’s *t*-test. The patency rates were estimated with Kaplan-Meier survival analysis, and the groups were compared using the log-rank test. Data were analyzed using the Statistical Package for the Social Sciences software version 25.0 (IBM Corp, Armonk, New York, NY, United States). A *p* value of <0.05 was considered statistically significant.

## Results

### Baseline Characteristics

The baseline characteristics of the patients with ABC after LDLT using an RL graft are summarized in Table 1. The mean age of the patients was 55.2 ± 8.6 years, and 77.2% were male. The most common etiology of liver cirrhosis was hepatitis B virus (34/57, 59.6%), followed by alcoholic liver cirrhosis (8/57, 14%) and hepatitis C virus (7/57, 12.3%). The average Model for End-stage Liver Disease score was 16.2 ± 6.8, and the average Child-Pugh score was 7.8 ± 2.6. The number of ABO-compatible donors and recipients was 82.5% (47/57). The mean follow-up duration was 44.2 ± 1.7 months. The total number of biliary interventions performed during follow-up was 5.2 ± 3.4.

**TABLE 1:** Demographic and baseline characteristics of the study population.

Variables	(*n* = 57)
Age, mean ± SD, years	55.2 ± 8.6
Sex, *n* (%)	
Male	44 (77.2%)
Female	13 (22.8%)
Etiology of liver cirrhosis, *n* (%)	
Hepatitis B virus	34 (59.6%)
Alcoholic liver cirrhosis	8 (14.0%)
Hepatitis C virus	7 (12.3%)
Non-B and non-C hepatitis	3 (5.3%)
Autoimmune hepatitis	2 (3.5%)
Hepatitis B virus with alcoholic liver cirrhosis	1 (1.8%)
Primary biliary cirrhosis	1 (1.8%)
BMI, mean ± SD, $\text{kg}/{\text{m}}^{2}$	23.4 ± 3.3
MELD score, mean ± SD	16.2 ± 6.8
Child-Pugh score, mean ± SD	7.8 ± 2.6
ABO compatibility between donor and recipient, *n* (%)	
Compatible pairs	47 (82.5%)
Incompatible pairs	10 (17.5%)
Follow-up duration, mean ± SD, month	44.2 ± 1.7
Total interventions during the follow-up period, mean ± SD	5.2 ± 3.4
Donor age, mean ± SD, years	35.5 ± 12.2
Donor hepatectomy type, *n* (%)	
Laparoscopic method	33 (57.9%)
Open method	24 (42.1%)
Graft bile duct	
Number of variations, *n* (%)	17 (29.8%)
Number of openings, mean ± SD	1.7 ± 0.7
Size, mean ± SD, mm	4.8 ± 2.1
Bile duct anastomosis—TTR method, *n* (%)	36 (63.2%)
Intraoperative biliary drainage, *n* (%)	7 (12.3%)
Intraoperative hepatic artery problem, *n* (%)	6 (10.5%)
Postoperative hepatic artery occlusion, *n* (%)	3 (5.3%)
Postoperative bleeding, *n* (%)	8 (14.0%)
Duration to initial intervention, mean ± SD, month	
All interventions	9.0 ± 8.6
RAD intervention	10.6 ± 9.0
RPD intervention	10.9 ± 9.2
Clinical manifestation, *n* (%)	
LFT abnormality	52 (91.2%)
Itching	14 (24.6%)
Jaundice	9 (15.8%)
Fever	5 (8.8%)
Abdominal pain	5 (8.8%)
Laboratory findings before the initial intervention, mean ± SD	
WBC, 10³/μL	5.8 ± 2.7
CRP, mg/dL	1.7 ± 4.0
Total bilirubin, mg/dL	2.3 ± 2.7
Direct bilirubin, mg/dL	1.6 ± 2.2
ALP, IU/L	305.0 ± 198.2
GGT, IU/L	569.8 ± 593.3
AST, IU/L	95.7 ± 77.6
ALT, IU/L	153.1 ± 170.8

SD, standard deviation; BMI, body mass index; MELD, model for end-stage liver disease; TTR, tailored telescopic reconstruction; RAD, right anterior hepatic duct; RPD, right posterior hepatic duct; LFT, liver function test; WBC, white blood cell; CRP, C-reactive protein; ALP, alkaline phosphatase; GGT, gamma-glutamyl transferase; AST, aspartate aminotransferase.

The mean age of the liver donors was 35.5 ± 12.2 years, with 33 (57.9%) laparoscopic donor hepatectomies and 24 (42.1%) open donor hepatectomies performed. There were 17 (29.8%) cases with graft bile duct variation. The average number of bile duct openings was 1.7 ± 0.7, and the average bile duct diameter was 4.8 ± 2.1 mm. Thirty-six (63.2%) patients underwent DD biliary reconstruction using the TTR method (10), and seven (12.3%) patients underwent intraoperative biliary drainage. Intraoperative hepatic artery complications, postoperative hepatic artery occlusion, and postoperative bleeding were noted in six (10.5%), three (5.3%), and eight (14%) cases, respectively. The mean period from the LDLT to the first intervention performed for ABC was 9 ± 8.6 months. Among patient diagnosed with ABC, the most common clinical manifestation was abnormal blood liver function test results (52/77, 91.2%), followed by itching (14/57, 24.6%), jaundice (9/57, 15.8%), fever (5/57, 8.8%), and abdominal pain (5/57, 8.8%). The mean total bilirubin and C-reactive protein levels prior to the first intervention for ABC were 2.3 ± 2.7 mg/dl and 1.7 ± 4.0 mg/dl, respectively.

### Clinical Outcomes

The clinical outcomes of both interventions are summarized in Table 2. These results were analyzed according to whether the interventions were performed on the RAD or RPD.

**TABLE 2:** Clinical outcomes of biliary interventions in the study population.

	RAD Involvement (*n* = 44)			RPD Involvement (*n* = 51)		
	ERCP (*n* = 29)	PTBD (*n* = 15)	*p* value	ERCP (*n* = 21)	PTBD (*n* = 30)	*p* value
Type of biliary complication, *n* (%)						
Stricture	28 (96.6%)	11 (73.3%)		17 (81%)	23 (76.7%)	
Leakage	0 (0%)	1 (6.7%)		3 (14.3%)	3 (10%)	
Stricture with leakage	1 (3.4%)	3 (20%)		1 (4.7%)	4 (13.3%)	
Success rate, *n* (%)	24 (82.8%)	10 (66.7%)	0.27	10 (47.6%)	21 (70%)	0.15
Total interventions during the follow-up period, mean ± SD	6.7 ± 3.6	4.5 ± 2.2	0.03	4.6 ± 3.1	6 ± 3.6	0.17
Patency period, mean ± SD, days	115 ± 40.3	126 ± 28.3	0.27	176 ± 29.0	283 ± 74.6	0.75

RAD, right anterior hepatic duct; RPD, right posterior hepatic duct; ERCP, endoscopic retrograde cholangiopancreatography; PTBD, percutaneous transhepatic biliary drainage; SD, standard deviation.

Among the patients with RAD involvement, ERCP and PTBD were attempted in 29 and 15 cases, respectively. Among the patients who underwent ERCP, 28 (96.6%) patients had anastomosis site stricture, and 1 (3.4%) patient had stricture with leakage. Among the patients who underwent PTBD, 11 (73.3%), 1 (6.7%), and 3 (20.0%) patients had anastomosis stricture, leakage, and stricture with leakage, respectively. The success rates of ERCP and PTBD were 82.8% and 66.7%, respectively. There was no significant difference in the success rate between the two groups (*p* = 0.27); however, the ERCP group underwent significantly more interventions during the follow-up period than the PTBD group (6.7 ± 3.6 vs. 4.5 ± 2.2, *p* = 0.03). Among the patients who underwent ERCP, the patency rates at 6, 12, and 24 months were 27.6%, 10.3%, and 3.4%, respectively. In contrast, the patency rates at 6, 12, and 24 months in the PTBD group were 40%, 33.3%, and 13.3%, respectively. There was no significant difference in the median patency period (115 ± 40.3 vs. 126 ± 28.3 days; *p* = 0.27) between the two groups.

Among the patients with RPD involvement, ERCP was performed for 17 (81%), 3 (14.3%), and 1 (4.7%) cases of anastomosis site stricture, leakage, and stricture with leakage, respectively, whereas PTBD was performed for 23 (76.7%), 3 (10%), and 4 (3.3%) cases of anastomosis site stricture, leakage, and stricture with leakage, respectively. The success rates of ERCP and PTBD were 47.6% and 70%, respectively; however, the difference was not statistically significant (*p* = 0.15). There was no significant difference in the total number of interventions during the follow-up period between both groups (4.6 ± 3.1 vs. 6 ± 3.6; *p* = 0.17). The patency rates of ERCP at 6, 12, and 24 months were 47.6%, 28.6%, and 28.6%, respectively, whereas the patency rates of PTBD at the same timepoints were 60%, 40%, and 19.4%, respectively. There was no significant difference in the median patency period between the two groups (176 ± 29 vs. 283 ± 74.6 days; *p* = 0.75).

### Comparison of Variables Affecting Intervention Success

We analyzed the variables that affected the success of ERCP and PTBD in the RAD and RPD involvement groups. Among the patients with RAD involvement, ERCP was significantly more likely to fail in patients with graft bile duct variations than in patients without variations (80% vs. 16.7%, *p* = 0.013). The R-G angle was also significantly greater in the group where ERCP failed than in the group where ERCP was successful (54.8 ± 24.2° vs. 28.8 ± 18.6°; *p* = 0.012). In comparison, there were no significant differences in the above variables when PTBD was performed for ABC of the RAD (Table 3). However, the success rate of PTBD tended to decrease as the number of bile duct anastomoses increased (*p* = 0.083). There were also no statistically significant differences in the variables when both ERCP and PTBD were performed for ABC of the RPD (Table 3).

**TABLE 3:** Comparison of clinical variables with the intervention results across the RAD and RPD involvement groups.

	RAD Involvement (*n* = 44)						RPD Involvement (*n* = 51)					
	ERCP in RAD (N = 29)			PTBD in RAD (*n* = 15)			ERCP in RPD (*n* = 21)			PTBD in RPD (*n* = 30)		
	Success (*n* = 24)	Failure (*n* = 5)	*p* value	Success (*n* = 10)	Failure (*n* = 5)	*p* value	Success (*n* = 10)	Failure (*n* = 11)	*p* value	Success (*n* = 21)	Failure (*n* = 9)	*p* value
Graft bile duct variation, *n* (%)	4 (16.7%)	4 (80%)	0.013	3 (30%)	3 (60%)	0.33	2 (20%)	2 (18.2%)	1.00	8 (38.1%)	3 (33.3%)	1.00
Hepatic artery complications, *n* (%)	2 (8.3%)	0 (0%)	1.00	3 (30%)	0 (0%)	0.51	0 (0%)	2 (18.2%)	0.48	3 (14.3%)	1 (11.1%)	1.00
Bile duct anastomosis—TTR method, *n* (%)	15 (62.5%)	4 (80%)	0.63	6 (60%)	4 (80%)	0.60	7 (70%)	4 (36.3%)	0.20	16 (76.2%)	5 (55.6%)	0.39
Donor surgical approach, *n* (%)						1.00			0.18			0.43
Laparoscopic method	9 (37.5%)	3 (60%)	0.62	7 (70%)	3 (60%)		5 (50%)	9 (81.8%)		9 (42.9%)	6 (66.7%)	
Open method	15 (62.5%)	2 (40%)		3 (30%)	2 (40%)		5 (50%)	2 (18.2%)		12 (57.1%)	3 (33.3%)	
Intraoperative drainage, *n* (%)	2 (8.3%)	0 (0%)	1.00	2 (20%)	2 (40%)	0.56	1 (10%)	1 (9.1%)	1.00	3 (14.3%)	1 (11.1%)	1.00
Number of bile ducts, *n* (%)			0.72			1.00			1.00			0.57
One	10 (41.7%)	2 (40%)		2 (20%)	2 (40%)		3 (30%)	6 (54.5%)		6 (28.6%)	4 (44.4%)	
Two	12 (50%)	2 (40%)		6 (60%)	1 (20%)		7 (70%)	3 (27.3%)		11 (52.4%)	4 (44.4%)	
Three	2 (8.3%)	1 (20%)		2 (20%)	2 (40%)		0 (0%)	2 (18.2%)		4 (19%)	1 (11.1%)	
Number of bile duct anastomoses, *n* (%)			0.553			0.083			0.635			0.477
One	21 (87.5%)	4 (80.0%)		8 (80.0%)	2 (40.0%)		8 (80.0%)	7 (63.6%)		18 (85.7%)	6 (66.7%)	
Two	3 (12.5%)	1 (20.0%)		2 (20.0%)	2 (40.0%)		2 (20.0%)	4 (36.4%)		2 (9.5%)	3 (33.3%)	
Three	0	0		0	1 (20.0%)		0	0		1 (4.8%)	0	
Bile duct size, mean ± SD, mm	4.7 ± 1.8	6.6 ± 4.1	0.37	4.5 ± 1.8	2.8 ± 1.5	0.13	5.7 ± 1.7	5.45 ± 2.1	0.76	3.74 ± 1.26	4.56 ± 2.02	0.20
Angle between graft and recipient bile ducts, mean ± SD, °	28.8 ± 18.6	54.8 ± 24.2	0.012	47.5 ± 25.8	44.7 ± 26.8	0.85	90.8 ± 41.1	100.13 ± 25.7	0.55	99.62 ± 23.20	99.79 ± 15.01	0.98

RAD, right anterior hepatic duct; RPD, right posterior hepatic duct; ERCP, endoscopic retrograde cholangiopancreatography; PTBD, percutaneous transhepatic biliary drainage; TTR, tailored telescopic reconstruction; SD, standard deviation.

### Prediction of Intervention Failure Using the R-G Angle

**FIGURE 3:**
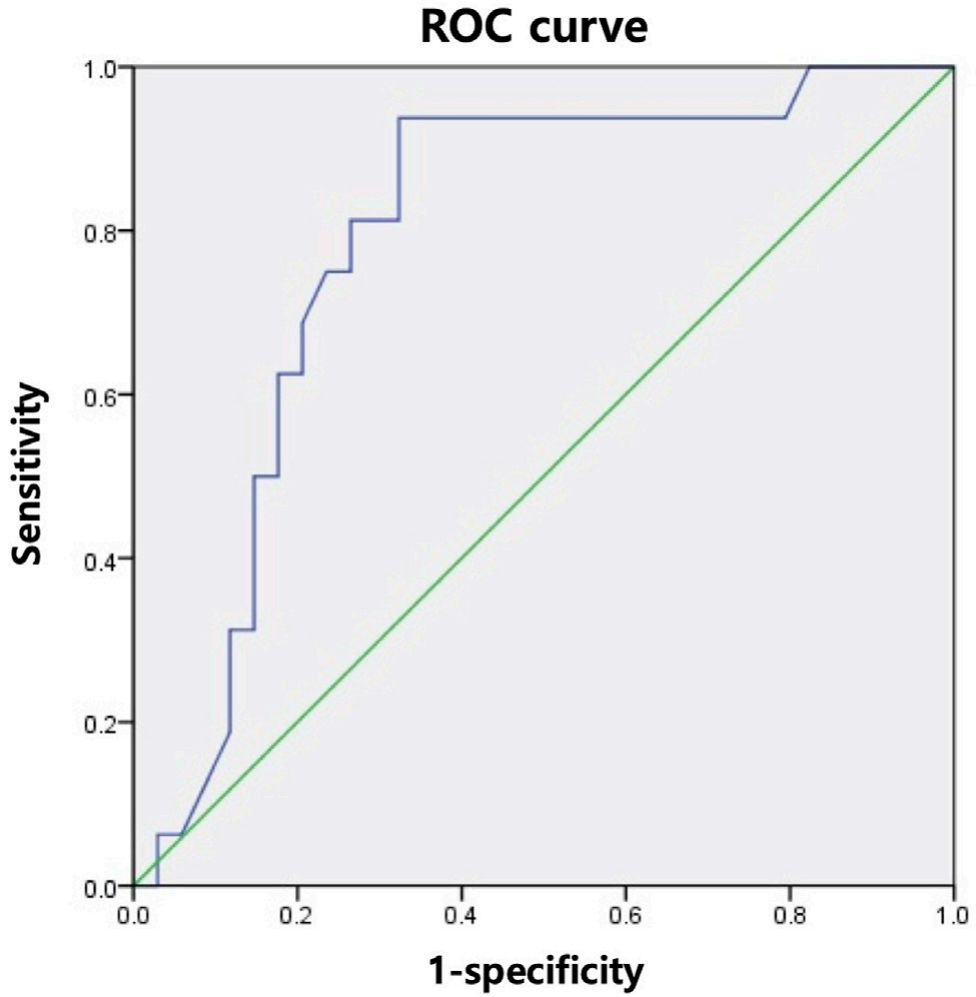
A receiver operating characteristic curve analysis for the angle between the graft and recipient bile ducts and likelihood of endoscopic retrograde cholangiopancreatography failure. ROC, receiver operating characteristic.

To predict the likelihood of intervention failure, we analyzed the R-G angles of the RAD and RPD groups for each intervention with the receiver operating characteristic (ROC) curve. An optimal cut-off point was calculated using Youden’s index.(11) The ROC curve analysis in Figure 3 demonstrates that when the R-G angle was greater than 47.5°, ERCP was more likely to fail (sensitivity, 93.8%; specificity, 67.6%). The ROC curve analysis for PTBD showed no statistically significant R-G angle cut-off value.

## Discussion

Our study demonstrated two novel features. First, we compared the clinical outcomes between ERCP and PTBD as the primary treatment for ABC after LDLT. Despite the advancements in biliary reconstruction techniques, biliary complications after LT are major and unavoidable complications. Non-surgical alternatives, such as endoscopic and radiologic interventional treatment, are increasingly becoming the treatment of choice for biliary complications after LT. Endoscopic treatment is generally performed as the first-line treatment, and PTBD is performed as a rescue treatment for when endoscopic interventions fail. Most studies consider ERCP as the safer and more convenient alternative, and ERCP is associated with fewer complications than PTBD. Only one study has compared the clinical outcomes of ERCP and PTBD (5, 12). This study demonstrated that the success and patency rates of ERCP and PTBD were similar, but the number of repeated procedures was higher in PTBD. The study also presented several disadvantages associated with PTBD, such as incidental PTBD removal, catheter associated pain, bile leakage around the catheter, and infection. The clinical outcomes in our study correlated well with the results of previous studies. The success rate, total number of interventions, and patency rate were similar between the ERCP and PTBD groups with RPD involvement. However, concerning RAD involvement, the total number of interventions and short-term patency rates were superior in the PTBD group than in the ERCP group. Therefore, when biliary intervention was attempted under specific patient conditions, including patients with RAD involvement who desired fewer interventions or a shorter follow-up period, our data demonstrated that PTBD was a good first-line option for ABC.

Second, our study highlighted several clinical criteria that should be considered when selecting between ERCP and PTBD for the treatment of ABC in the RAD or RPD. LDLT is currently performed in countries with low deceased donor availability, which are mainly comprised of Asian countries, such as South Korea (13–15). LDLT typically uses an RL graft, but this can have multiple bile duct openings, which is a risk factor for ABC (1, 16, 17). Performing simultaneous bilateral bile duct drainage of multiple openings with either ERCP or PTBD may provide more effective long-term benefits in patients with ABC after LDLT than unilateral biliary drainage (4). We further analyzed the factors affecting the success rates of ERCP and PTBD for ABC with RAD and RPD involvement. Previous studies proposed that hepatic artery stenosis and stricture morphology affect the success of endoscopic management (3, 18, 19). We examined several intraoperative technical factors associated with stricture morphology and intraoperative hepatic artery complications, such as whether TTR of the bile duct or laparoscopic donor hepatectomy was the superior method (10, 15).

Among the patients with RAD involvement, the presence or absence of graft bile duct variation affected the success of ERCP. Variations in the right hepatic duct are determined by the location of the RPD, and RPD variation of the RL graft increases the likelihood for ABC (6, 20). Our results contrasted with published literature, as our study demonstrated that RAD involvement was more associated with ABC than RPD involvement. In single-centers, a multidisciplinary approach with surgeons, radiologists, and interventionists should be considered when postoperative complications are expected in LT recipients. In particular, when anatomic complications are likely due to RPD involvement, PTBD is preferred over ERCP. Our study followed a retrospective design and examined a small sample size. Therefore, selection bias was possible, and the presence or absence of graft bile duct variation in RPD involvement may not significantly affect the success of ERCP. While our results suggested that PTBD was more effective for patients with RAD involvement, further large-scale studies are needed to confirm the clinical significance of this result.

Our study also indicated that large R-G angles increase the likelihood of failure of ERCP with RAD involvement. The mean R-G angles for successful and unsuccessful procedures were 28.8 ± 18.6° and 54.8 ± 24.2°, respectively. Our fluoroscopic findings were consistent with the results of previous studies, which demonstrated that acute angulation increased the likelihood of failure of ERCP (4, 21). Our ROC analysis (Figure 3) suggested that an R-G angle (in either the RAD or RPD) greater than 47.5° was significantly associated with ERCP failure (sensitivity, 93.8%; specificity, 67.6%). As shown in Table 2, while there was no statistically significant difference in the success rates between ERCP and PTBD, the success rate of ERCP with RAD involvement was 82.8%, which was higher than that of PTBD. In contrast, the success rate of ERCP with RPD involvement fell to 47.6%, which was lower than that of PTBD. Therefore, when bile duct angulation is considered alone (in either the RAD or RPD), PTBD may be the superior first-line treatment of choice for biliary drainage in ABC compared to ERCP when the R-G angle is greater than 47.5°.

Of the 57 patients in our study, 15 underwent biliary interventions for concomitant RAD and RPD involvement. Three, six, one, and five patients underwent bilateral ERCP (E/E group), ERCP and PTBD (E/P group), PTBD and ERCP (P/E group), and bilateral PTBD (P/P group), respectively. The success rates in the RAD and RPD were both 100% in the E/E group, 100% and 50% in the E/P group, both 0% in the P/E group, and both 80% in the P/P group. While it was difficult to determine whether there was a statistically significant difference among these results, the success rate seemed to be higher when the same intervention was performed for both the RAD and RPD. Further studies are needed to accurately evaluate the suitability of combining ERCP and PTBD.

This study has several limitations. First, our study design may have been prone to selection bias, because it was a retrospective, single-center cohort with a small sample size. Second, while we analyzed clinical outcomes and influential factors based on RAD and RPD involvement, performing multiple procedures in a clinical setting may affect the results. Third, ABC was diagnosed based on radiologic findings, which might have been influenced by the researcher’s subjectivity. ABC can be difficult to differentiate from non-ABC. Fourth, several interventionists performed the ERCP and PTBD procedures, and differences in operative technique might have affected the final outcomes. Fifth, we limited our study participants to patients newly diagnosed with ABC following LDLT, but previous studies have shown that 12–35.6% of patients who undergo LDLT develop biliary complications, and biliary complications recur in approximately 20% (5, 9). In addition, non-anastomotic biliary complications (non-ABC) are diagnosed in 5–15% of patients, and non-ABC are associated with high recurrence rates and poor graft prognosis (22). Therefore, it is necessary to conduct further studies with a larger sample size including patients with recurrent biliary complications and non-ABC, as these account for a large proportion of patients with biliary complications.

Despite these limitations, our study was significant, clearly demonstrating that either ERCP or PTBD may be used as first-line treatment options for ABC after LDLT. Our study was also the first to attempt classification and evaluation of these interventions based on RAD and RPD involvement.

In conclusion, both ERCP and PTBD were appropriate first-line treatments for ABC after LDLT. Several factors must be considered when determining the optimal treatment for ABC, and the success of ERCP and PTBD may be influenced by whether the RAD and/or RPD are involved. Specifically, PTBD is recommended in patients with RAD involvement when there is graft bile duct variation and in patients with either RAD or RPD involvement where the angle between the recipient and graft bile ducts is greater than 47.5°.

## Data Availability

The raw data supporting the conclusion of this article will be made available by the authors, without undue reservation.

## Abbreviations

ABC, Anastomotic biliary complications; ERCP, Endoscopic retrograde cholangiopancreatography; LDLT, Living donor liver transplantation; PTBD, Percutaneous transhepatic biliary drainage; RAD, Right anterior hepatic duct; R-G angle, Angle between recipient bile duct and graft bile duct; RL, Right hemi-liver; RPD, Right posterior hepatic duct.
